# A comprehensive theoretical model for on-chip microring-based photonic fractional differentiators

**DOI:** 10.1038/srep14216

**Published:** 2015-09-18

**Authors:** Boyuan Jin, Jinhui Yuan, Kuiru Wang, Xinzhu Sang, Binbin Yan, Qiang Wu, Feng Li, Xian Zhou, Guiyao Zhou, Chongxiu Yu, Chao Lu, Hwa Yaw Tam, P. K. A. Wai

**Affiliations:** 1 State Key Laboratory of Information Photonics and Optical Communications, Beijing University of Posts and Telecommunications, P.O. Box 72 (BUPT), Beijing 100876, China; 2 Photonics Research Centre, Department of Electronic and Information Engineering, The Hong Kong Polytechnic University, Hung Hom, Kowloon, Hong Kong; 3 Department of Physics and Electrical Engineering, Northumbria University, Newcastle upon Tyne, NE1 8ST, United Kingdom; 4 Guangdong Provincial Key Laboratory of Nanophotonic Functional Materials and Devices, South China Normal University, 510006 Guangzhou, China; 5 Department of Electrical and Computer Engineering, University of Nebraska-Lincoln, Lincoln, Nebraska 68588, United States

## Abstract

Microring-based photonic fractional differentiators play an important role in the on-chip all-optical signal processing. Unfortunately, the previous works do not consider the time-reversal and the time delay characteristics of the microring-based fractional differentiator. They also do not include the effect of input pulse width on the output. In particular, it cannot explain why the microring-based differentiator with the differentiation order *n* > 1 has larger output deviation than that with *n* < 1, and why the microring-based differentiator cannot reproduce the three-peak output waveform of an ideal differentiator with *n* > 1. In this paper, a comprehensive theoretical model is proposed. The critically-coupled microring resonator is modeled as an ideal first-order differentiator, while the under-coupled and over-coupled resonators are modeled as the time-reversed ideal fractional differentiators. Traditionally, the over-coupled microring resonators are used to form the differentiators with 1 < *n* < 2. However, we demonstrate that smaller fitting error can be obtained if the over-coupled microring resonator is fitted by an ideal differentiator with *n* < 1. The time delay of the differentiator is also considered. Finally, the influences of some key factors on the output waveform and deviation are discussed. The proposed theoretical model is beneficial for the design and application of the microring-based fractional differentiators.

With the rapid development of high-speed communication and computing, all-optical signal processing becomes more and more important in overcoming the bandwidth limitation of conventional electronics^1^,^2^. The temporal photonic differentiator is a basic element which can implement the time derivative of the complex envelope of an input optical pulse. It has wide applications in pulse characterization, ultra-fast signal generation, and ultra-high-speed coding^3^,^4^,^5^,^6^,^7^,^8^. A number of schemes have been proposed to realize the all-optical temporal differentiation, among which the on-chip microring resonator (MR) has been considered as a promising candidate because of its compactness, maturity in fabrication, and opto-electronic integration^9^,^10^,^11^,^12^,^13^,^14^,^15^.

In addition to the integer-order differentiation, the concept of fractional derivative has also attracted much interest in image processing, automatic control, fluid flow, electromagnetic theory, electrical networks, and biomedical applications^16^,^17^,^18^,^19^,^20^,^21^,^22^. It has been recently demonstrated that fractional differentiation with the differentiation order 0 < *n *< 2 can be implemented by using the under- and over-coupled microring resonators^23^,^24^,^25^. Previous works use the transfer function of the microring resonator to simulate the output of the differentiator, but it is not explained how the response of a microring resonator can be fitted by an ideal fractional differentiator. Besides, these works are based on the incorrect assumption that a phase jump, which is larger or smaller than π across the resonance, can be obtained in the under- or over- coupled resonators. In fact, due to the limited bandwidth of the input pulse, the effective phase jump for the input is always smaller than π. As a result, there are a number of shortcomings in these works. Firstly, the time-reversal characteristic of the microring-based differentiators is ignored. The output of the resonator should be approximated by a time-reversed ideal fractional differentiator, not an ideal fractional differentiator as was done in the literature. Secondly, the time delay of the output is not considered. However, the dependence of the time delay on the differentiation order is crucial for a continuously tunable fractional differentiator. Thirdly, the effect of the input pulse width on the output of the microring-based differentiator is not included. Finally, the previous works cannot explain why the microring-based differentiator with *n* > 1 has larger output deviation than that with 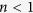, and why the microring-based differentiators cannot reproduce the three-peak output waveform of an ideal differentiator with 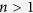. Thus, the design and application of the microring-based fractional differentiators are greatly limited.

In this paper, a comprehensive theoretical model for on-chip microring-based fractional differentiators is proposed. We investigate how the all-pass microring resonator can be fitted by an ideal fractional differentiator. Within the frequency range of the input pulse, the phase response of a microring resonator can be approximated by a three-segment polyline. The time-reversal characteristic and the time delay of the microring-based differentiator are considered. It is revealed that the critically-coupled microring resonator can be modeled as an ideal first-order differentiator, while the under-coupled and the over-coupled resonators can be modeled as the time-reversed ideal fractional differentiators. Traditionally, the over-coupled microring resonator is used to form the differentiator with 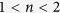. However, we demonstrate that smaller fitting error can be obtained if the over-coupled microring resonator is fitted by an ideal differentiator with 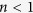 instead. Thus, since there are only two peaks in an ideal differentiated pulse with 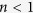, it can be explained that the microring-based differentiator with 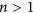 cannot reproduce the three-peak waveform of the ideal differentiated pulse. In addition to the output deviation induced by the resonator itself, the influences of key factors such as the divergence of the carrier from the resonant wavelength, input pulse width, and finite slope of the phase response across the resonance, on the output waveform and deviation are also discussed.

## Results

The previous works have not elaborated how the all-pass microring resonator is approximated by an ideal fractional differentiator, and the time delay characteristic of the microring-based differentiators is also neglected. Hence, a comprehensive theoretical model is proposed, and is validated by the output waveforms of the differentiators. By analyzing in the frequency domain, it is demonstrated that the microring-based fractional differentiator can be approximated by an ideal differentiator or a time-reversed ideal differentiator with a certain time delay. Furthermore, the intrinsic deviation from an ideal fractional-order differentiator is explored.

In this work, a microring with the radius *R* = 50 μm is used. The silicon waveguides have a cross section of 450 nm × 250 nm. A Gaussian pulse with the temporal full width at half-maximum (FWHM) of 50 ps is launched into the resonator as the input signal. The optical carrier is TM polarized at 1439.5 nm, which is at one of the resonant wavelengths of the microring resonator. While keeping the transmission coefficient of the coupling region at r = 0.83, the coupling state of the resonator can be adjusted by changing the round-trip amplitude transmission 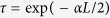, where *α* is the power loss coefficient and *L* is the circumference of the microring.

The microring resonator has three coupling states, namely under-, critical-, and over-coupling. The transmission and phase response for different coupling states have different features, which will affect the modeling of the microring-based fractional differentiators with different differentiation order *n*. Therefore, the model and the intrinsic deviation are discussed respectively according to the coupling state of the resonator. Here, we assume *τ* = 0.801 to form the typical *n* = 0.4-order differentiator as an example of the under-coupled microring resonators. For the over-coupled microring resonators, we assume *τ* = 0.846 to form the typical *n* = 1.5-order differentiator.

**Figure 1:**
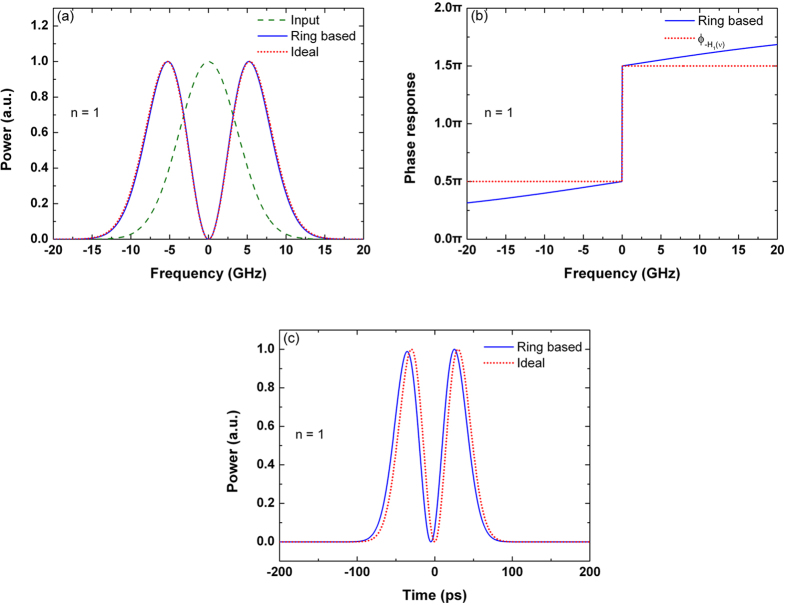
Differentiator based on the critically-coupled microring resonator. (**a**) Power spectra of the input Gaussian pulse, output differentiated signal, and ideal differentiated signal with the differentiation order *n* = 1. (**b**) Phase response of the microring-based differentiator and 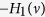. (**c**) Outputs of the microring-based differentiator and the ideal differentiator with *n* = 1.

The microring-based first-order differentiator is shown in Fig. 1. From the phase response shown in Fig. 1(b), the transfer function of the critically-coupled microring resonator can be modeled as 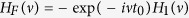, where 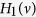 is the transfer function of the ideal first-order differentiator, and 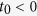 is a constant representing the time delay. Therefore, it can be deduced that the critically-coupled microring resonator can perform as a first-order differentiator with a negative time delay. This model is validated by Fig. 1(c), which shows the output waveforms of the critically-coupled microring resonator and the ideal first-order differentiator. From Fig. 1(c), the output waveform of the microring resonator is well described by 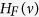.

The deviation of the output waveform is derived from the deviation of the intensity transmission and the phase response. Figure 1(a) shows a good agreement between the output power spectra of the microring-based differentiator and the ideal differentiator, which indicates that the intensity transmission of the critically-coupled resonator agrees well with that of the ideal first-order differentiator. Figure 1(b) shows that the phase response of the critically-coupled resonator accords well with 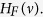


In order to quantitatively evaluate the output deviation, we define the average deviation 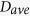 as


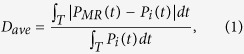


where 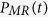 and 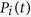 are the normalized output powers of the microring-based differentiator and the ideal differentiator, respectively. In this work, to ensure the successive output pulses do not overlap with each other, the time repetition period is chosen as 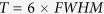, where FWHM is the full width at half maximum of the input pulses.

In Fig. 1(c), the deviation of the output waveform is 2.03%, which indicates that the first-order differentiator can be realized with very small deviation by the critically-coupled microring resonator.

**Figure 2:**
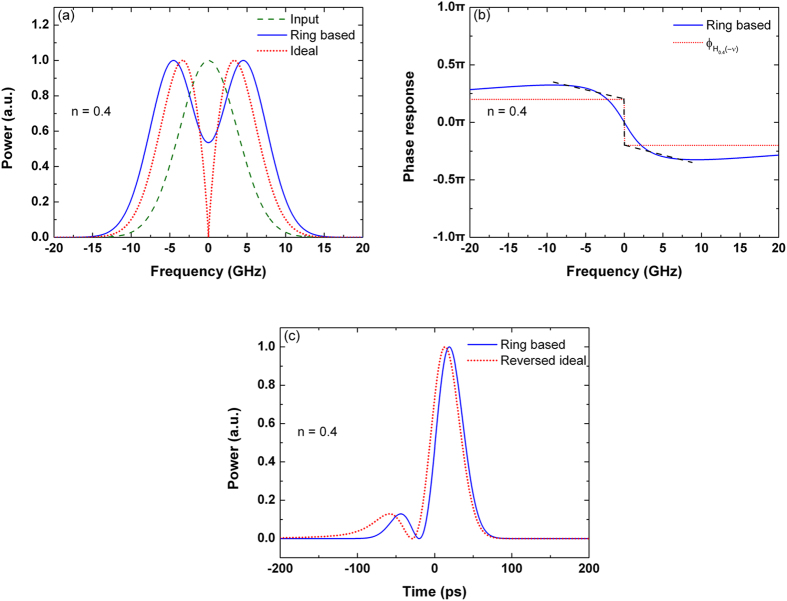
Differentiator based on the under-coupled microring resonator. (**a**) Power spectra of the input Gaussian pulse, output differentiated signal, and ideal differentiated signal with the differentiation order *n* = 0.4. (**b**) Phase response of the microring-based differentiator and 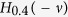. (**c**) Outputs of the microring-based differentiator and the time-reversed ideal differentiator with *n* = 0.4.

Figure 2 shows the 0.4-order differentiator based on the under-coupled microring resonator. The Fourier transform of the input Gaussian pulse is also a Gaussian function, where more than 90% of the pulse energy is concentrated in the frequency range from −7.5 to + 7.5 GHz. Hence, the output is mainly affected by the low frequency response of the resonator, and we will focus on fitting the response of the microring resonator with an ideal fractional differentiator within this frequency range. It is noteworthy that the power distribution in the frequency domain is affected by the pulse shape and pulse width in the time domain. Therefore, the modeling of the microring-based fractional differentiators is affected by the input pulse width, which will lead to a variation in the differentiation order. From the phase response shown in Fig. 2(b), the transfer function of the under-coupled microring resonator can be modeled as 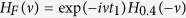, where 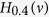 is the transfer function of the ideal differentiator with *n* = 0.4, and 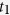 is positive inferred from the low frequency response of the resonator. Therefore, considering an ideal fractional differentiator with *n* < 1, the output of the under-coupled microring resonator can be approximated by reversing the output waveform of the ideal fractional differentiator and delaying it in time. This model is supported by Fig. 2(c), which shows the output waveforms of the microring resonator and the time-reversed ideal fractional differentiator.

In the time domain, the deviation of the microring-based differentiator is more pronounced near the side peak of the output waveform. From Fig. 2(c), the width of the side peak is smaller compared with the output of the ideal fractional differentiator. The deviation is caused by the frequency response of the microring resonator. As shown in Fig. 2(a), the microring does not provide sufficient attenuation at the carrier frequency, and results in an increase of the spectral width of the output pulse. Fig. 2(b) also shows that there are some apparent differences between the phase response of the resonator and the phase response of 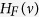. In particular, the slope of the phase response at the carrier frequency is much smaller than that of an ideal fractional differentiator. The effect of the finite slope of the phase response on the output deviation will be further discussed later. The deviation of the output waveform is 8.26%, as shown in Fig. 2(c).

**Figure 3:**
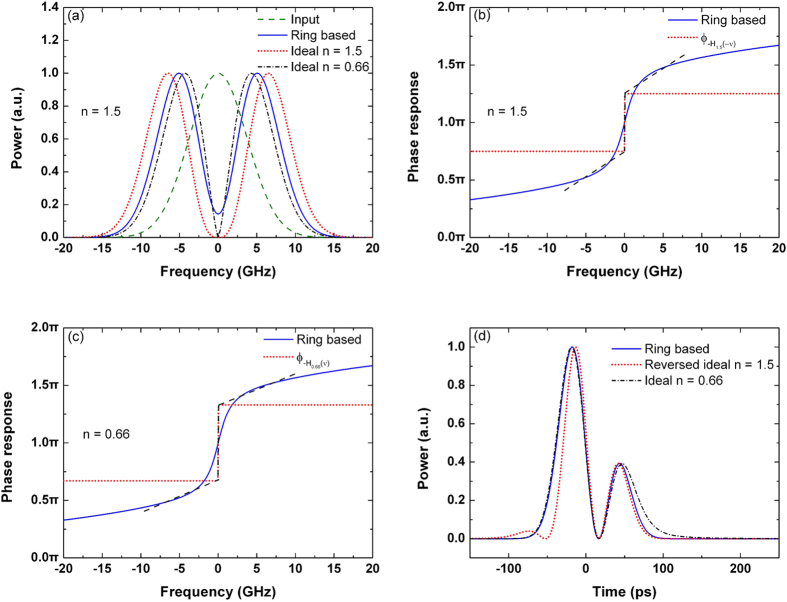
Differentiator based on the over-coupled microring resonator. (**a**) Power spectra of the input Gaussian pulse, output differentiated signal, and ideal differentiated signals with the differentiation orders of *n* = 1.5 and *n* = 0.66. (**b**) Phase response of the microring-based differentiator and 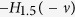. (**c**) Phase response of the microring-based differentiator and 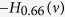. (**d**) Outputs of the microring-based differentiator (blue solid line), time-reversed ideal 1.5-order differentiator (red dotted line), and ideal 0.66-order differentiator (black dash-dotted line). The time delays are neglected.

The 1.5-order differentiator based on the over-coupled microring resonator is presented in Fig. 3. As the slope of the phase response across the resonance is much gradual, the variation of the phase shift is less than π in the frequency range of the input Gaussian pulse. From the phase response shown in Fig. 3(b), the transfer function of the over-coupled microring resonator can be modeled as 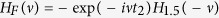, where 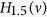 is the transfer function of the ideal differentiator with *n* = 1.5 and 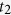 is negative inferred from the low frequency response of the resonator. Therefore, considering an ideal fractional differentiator with *n* > 1, the time-delayed output of the over-coupled microring resonator can be approximated by reversing the output waveform of the ideal fractional differentiator.

As proposed in^23^,^24^,^25^, the over-coupled microring resonator is normally used to form a fractional-order differentiator with *n* < 1 < 2. In fact, the over-coupled microring resonator can be also modeled as an ideal fractional differentiator with *n* < 1. As shown in Fig. 3(c), the transfer function of the over-coupled microring resonator is fitted by 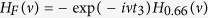, where 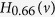 is the transfer function of the ideal differentiator with *n* = 0.66, and 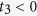 is a constant. Thus, the over-coupled microring resonator can be modeled as an ideal 0.66-order differentiator with a negative time delay.

From Fig. 3(a–c), it can be seen that smaller deviation of the frequency response can be obtained, when the over-coupled microring resonator is modeled as a differentiator with *n* < 1. This can be also observed by the output waveforms in the time domain, as shown in Fig. 3(d), where the time delays 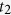 and 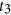 are neglected. The deviation of the output waveform is 21.84% when the microring-based resonator is fitted by the ideal 1.5-order differentiator, while the deviation is decreased to 7.64% if the resonator is fitted by the ideal 0.66-order differentiator. Compared with the output of the time-reversed ideal 1.5-order differentiator, the microring-based differentiator increases the width of the main peak, and cannot generate the small peak located at −70 ps. Compared with the output of the ideal 0.66-order differentiator, the minor deviation is due to a small decrease in the width of the minor peak. Therefore, it can be deduced that microring resonators are more suitable to form the differentiators with *n* < 1. Considering that there are only two peaks in an ideal differentiated pulse with *n* < 1, therefore it can be explained that the microring-based differentiators with *n* > 1 cannot reproduce the three-peak waveform of the ideal differentiated pulse.

## Discussion

As demonstrated above, the output deviation of the microring-based differentiator is affected by the differentiation order. In addition, the output waveform is also affected by other factors such as the carrier wavelength, input pulse width, and finite slope of the phase response at the resonant wavelength. In this section, the effects of these factors on the output waveform and the output deviation will be explored.

**Figure 4:**
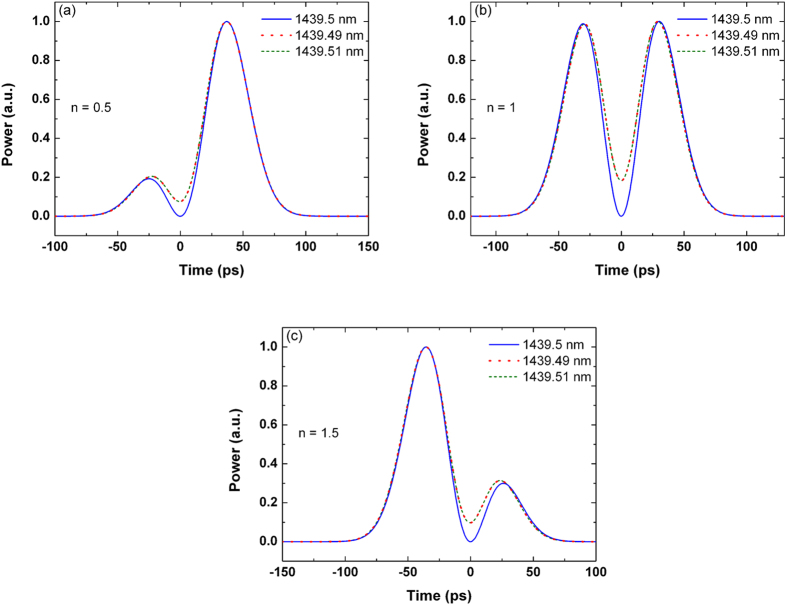
Outputs of the microring-based differentiators with (a) *n* = 0.5, (b) *n* = 1, and (c) *n* = 1.5 for various carrier wavelengths. The resonant wavelength of the microring resonator is 1439.5 nm.

The resonant wavelength is determined by the effect refractive index and the perimeter of the microring. In the microring-based differentiator, the optical carrier should coincide with one of the resonant wavelengths. However, the resonant wavelengths may be shifted by many factors, such as the ambient temperature, the optical intensity of the intracavity light-waves, and the bias voltage applied to the silicon microring resonator^24^. We study the effect of the wavelength mismatch by keeping the resonant wavelength constant and shifting the carrier wavelength by ± 0.01 nm. As shown in Fig. 4, when the resonant wavelength is 1439.5 nm, the corresponding carrier wavelength is changed from 1439.5, to 1439.49, and to 1439.51 nm. When the carrier and the resonant wavelength are mismatched, it can be seen that the optical power does not drop to zero between the two peaks of the output waveform. Hence, the deviation when compared with the ideal fractional differentiator will increase in the notch part of the output pulse.

**Figure 5:**
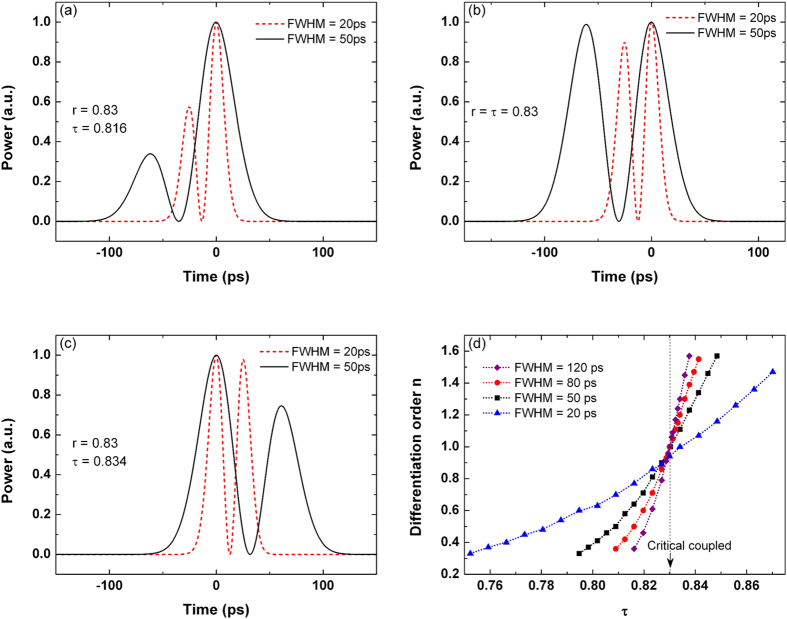
Output waveform of the differentiator for different input pulse widths based on (a) under-coupled resonator, (b) critically-coupled resonator, and (c) over-coupled resonator. (**d**) Differentiation order as a function of τ for different input pulse widths.

The differentiation order of the microring-based differentiator will be altered when the input pulse width is varied. As seen from Fig. 5(b), for the critically-coupled microring resonator, the first peak of the output will be decreased if the input FWHM decreases from 50 to 20 ps. In Fig. 5(a,c), for the under-coupled and the over-coupled microring resonators with *τ* = 0.816 and *τ* = 0.834 respectively, the minor peak of the output will be increased if the input FWHM decreases. Figure 5(d) shows the differentiation order versus τ for different input pulse widths. The output waveforms of the resonator for different *τ* are fitted with the ideal fractional differentiators using the least mean square (LMS) principle. We note that if the input pulse width decreases, the differentiation order will increase for the under-coupled microring resonators, but decrease for the over-coupled microring resonators. For the critically-coupled microring resonator used in our work, the differentiation order maintains at *n* = 1 for all the input pulses with FWHM ≥ 50 ps. However, the differentiation order will decrease if the input pulse width is decreased to FWHM = 20 ps.

**Figure 6:**
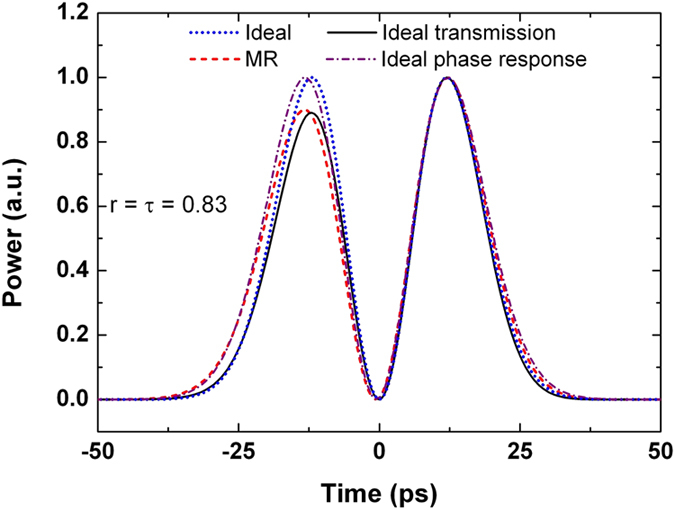
Output waveforms of four optical filters: the ideal first-order differentiator, critically-coupled microring resonator, combination of the transmission of the ideal differentiator and the phase response of the resonator, and combination of the transmission of the resonator and the phase response of the ideal differentiator. The input pulse width is FWHM = 20 ps.

To evaluate whether the intensity transmission or the phase response is more critical to the output waveform when the input pulse width is varied, two hypothetical differentiators are simulated. They are (i) the intensity transmission of the critically-coupled resonator with the phase response of the ideal first-order differentiator, and (ii) the phase response of the critically-coupled resonator with the intensity transmission of the ideal first-order differentiator. As shown in Fig. 6, for an input pulse with FWHM = 20 ps, the deviation of the output from the ideal first-order differentiator is significantly diminished in (i), while the output deviation cannot be effectively diminished in (ii). Therefore, it can be inferred that the output variation is mainly caused by the deviation of the phase response, rather than the amplitude response of the microring resonator from the ideal.

**Figure 7:**
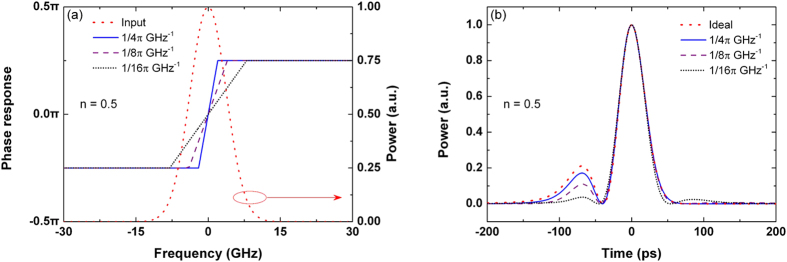
(**a**) Phase response and (**b**) corresponding output of the ideal 0.5-order differentiator except with the limited slope in the phase response. The power spectrum of the input Gaussian pulse with FWHM = 50 ps is also plotted in (**a**).

**Figure 8:**
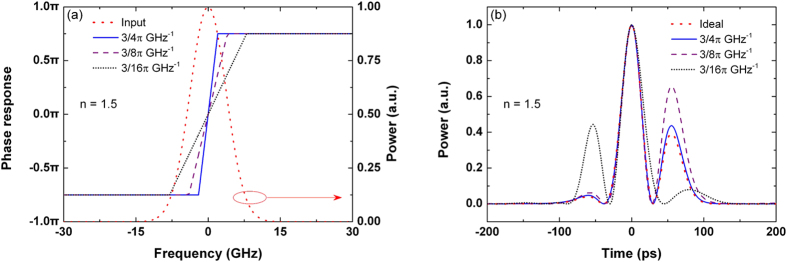
(**a**) Phase response and (**b**) corresponding output of the ideal 1.5-order differentiator except with the limited slope in the phase response. The power spectrum of the input Gaussian pulse with FWHM = 50 ps is also plotted in (**a**).

Compared with the ideal fractional differentiator, the slope of the phase response on the resonance is finite for under-coupled and over-coupled microring resonators. To study the effect of the finite slope on the output waveform, we decrease the slope in the phase response of the ideal differentiators at the resonant wavelength, as shown in Fig. 7(a) and 8(a). The corresponding output waveforms are simulated and depicted in Fig. 7(b) and 8(b). We note that the output deviation increases when the slope decreases. The output deviation is more pronounced at the side peak. For the 0.5-order differentiator, the output deviation increases from 4.56 to 16.53% when the slope decreases from 1/4*π* to 1/16*π* GHz^−1^, as shown in Fig. 7(b). For the 1.5-order differentiator, the output deviation increases from 3.97 to 21.18% when the slope decreases from 3/4*π* to 3/8*π* GHz^−1^. The position of the side peak also changes in the 1.5-order differentiated pulse when the slope is less than 3/16*π* GHz^−1^, as shown in Fig. 8(b).

## Methods

### Frequency response of the ideal and the microring-based photonic fractional differentiators

**Figure 9:**
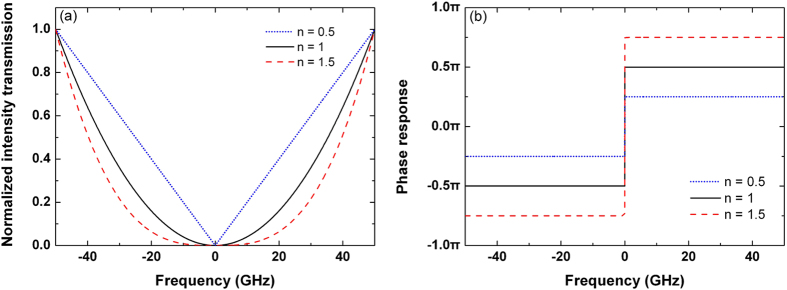
(**a**) Intensity transmission and (**b**) phase response of the ideal fractional differentiators with various differentiation orders *n*.

For an input signal *x*(*t*), the output of the *n*-th order differentiator is 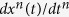. The ideal fractional-order differentiator can be considered as an optical filter. Its transfer function 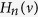 can be described by^26^,^27^,^28^.


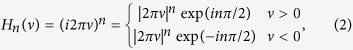


where 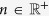, 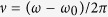 is the baseband frequency, 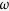 is the optical frequency, and 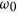 is the carrier frequency. As shown in Fig. 9(a), the intensity transmission of the ideal fractional differentiator is 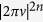, which is defined as the square of the amplitude response. The intensity transmission curves are symmetrical with respect to *v* = 0. Especially for *n* = 0.5, the intensity transmission is linear with respect to the baseband frequency on both sides of its symmetry axis. Besides, the bandwidth of the notch is increased with the increase of the differentiation order. The transmission is zero when the baseband frequency decreases to zero, which indicates that the optical signal will be attenuated completely at the carrier frequency. As shown in Fig. 9(b), the phase response has two opposite values, and there is a phase jump of *nπ* across 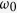. The phase response is positive and negative when *v* > 0 and *v* < 0, respectively.

**Figure 10:**
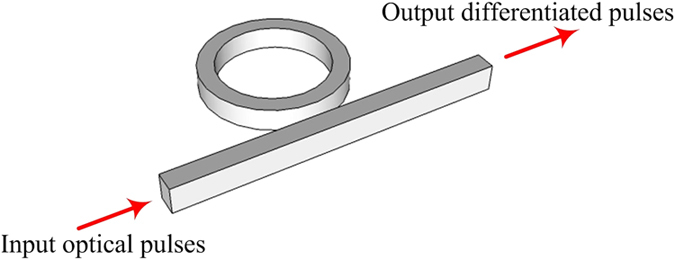
Schematic illustration of the fractional differentiator based on an all-pass microring resonator.

The transfer function of the ideal fractional differentiator can be approximately implemented by an all-pass microring resonator, the structure of which is illustrated in Fig. 10. The intensity transmission *T* and the phase response Φ of the all-pass microring resonator are given by^29^,^30^,^31^.


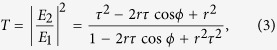



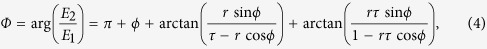


where *r* is the transmission coefficient of the coupling region, *ϕ* = *βL* is the round-trip phase shift with the circumference *L* and the propagation constant *β*, and 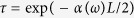 is the round-trip amplitude transmission with the power loss coefficient *α*.

**Figure 11:**
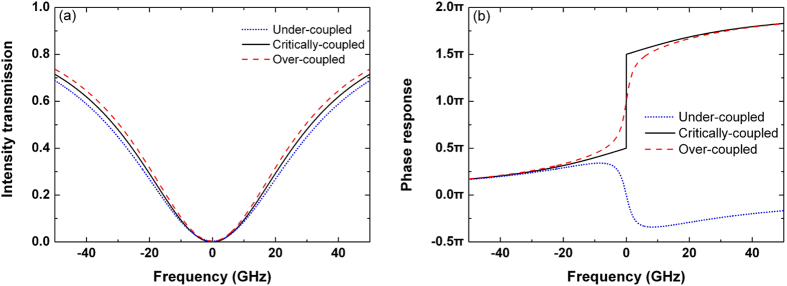
(**a**) Intensity transmission and (**b**) phase response of the microring resonator with different coupling states.

The microring resonator has three coupling states, namely under-coupling with 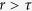, critical-coupling with 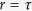, and over-coupling with 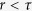
29. When *τ* is varied and *r* is kept constant, Fig. 11 shows the baseband intensity transmission and the baseband phase response of the under-coupled, critically-coupled, and over-coupled microring resonators. In Fig. 11(a), the bandwidth is increased with the decrease of 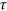. In Fig. 11(b), the critically-coupled microring resonator can generate a phase jump of *π* across the resonance. In this work, the carrier is launched at the resonant wavelength. Therefore, the first-order differentiator can be approximately implemented when the microring resonator is operated at the critical-coupling condition. Likewise, as a phase variation different from *π* can be achieved using the under-coupled and the over-coupled microring resonators, the fractional-order differentiator with *0* < *n* < 2 can be realized by changing the coupling state of the microring resonator^24^.

## Conclusion

In summary, a comprehensive theoretical model of the microring-based fractional differentiator is proposed. Compared with the previous reports in which the time-reversal characteristic is ignored, it is demonstrated that the under-coupled and the over-coupled microring resonators can be modeled as the time-reversed ideal fractional differentiators with the differentiation order *n* < 1 and *n* > 1, respectively. We also show that smaller fitting error can be obtained if the over-coupled microring resonator is fitted by an ideal differentiator with *n* < 1 instead. The time delay characteristic is also investigated. The influences of some key factors on the output waveform and deviation are also discussed. When the carrier and the resonant wavelength are mismatched, the deviation will be increased in the notch part of the output pulse. The variation of the input pulse width may cause a change in the differentiation order, where the phase response of the microring resonator plays an important role. The finite slope on the resonance in the phase response will lead to an amplitude deviation of the side peak in the output waveform. If the slope is further decreased, the position of the side peak will also be changed. The proposed theoretical model would be beneficial for the design, adjustment, and implementation of the on-chip microring-based fractional differentiators.

## Additional Information

**How to cite this article**: Jin, B. *et al.* A comprehensive theoretical model for on-chip microring-based photonic fractional differentiators. *Sci. Rep.* **5**, 14216; doi: 10.1038/srep14216 (2015).
